# Sensor Array Based Determination of Edman Degradated Amino Acids Using Poly(*p*‐phenyleneethynylene)s

**DOI:** 10.1002/chem.202001262

**Published:** 2020-05-29

**Authors:** Hao Zhang, Benhua Wang, Kai Seehafer, Uwe H. F. Bunz

**Affiliations:** ^1^ Organisch-Chemisches Institut Ruprecht-Karls-Universität Heidelberg Im Neuenheimer Feld 270 69120 Heidelberg Germany; ^2^ College of Chemistry & Chemical Engineering Central South University Changsha 410083 Hunan Province China; ^3^ Centre for Advanced Materials Im Neuenheimer Feld 225 69120 Heidelberg Germany

**Keywords:** amino acids, anions, Edman degradation, polymers, sensor arrays

## Abstract

A cross‐reactive optical sensor array based on poly(*p*‐phenyleneethynylene)s (PPEs) determines Edman degraded amino acids. We report a sensor array composed of three anionic PPEs **P1–P3**, and their electrostatic complexes with metal ions (Fe^2+^, Cu^2+^, Co^2+^). We recorded distinct fluorescence intensity response patterns as “fingerprints” of this chemical tongue toward standard phenylthiohydantoin (PTH) amino acids—degradation products of the Edman process. These “fingerprints” were converted into canonical scores by linear discrimination analysis (LDA), which differentiates all of the PTH‐amino acids. This array discriminates PTH‐amino acid residues degraded from an oligopeptide through Edman sequencing. This approach is complementary to chromatography approaches which rely on mass spectrometry; our array offers the advantage of simplicity.

## Graphical abstract

**An approach** for the determination of Edman degradation products using PPE‐based sensor array is described. The nine‐element sensor array composed of three anionic PPEs, together with the corresponding electrostatic complexes with metal cations was readily utilized for identification and determination of PTH‐amino acids (see figure).

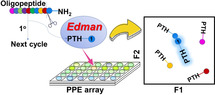

Herein, we report a sensor array composed of three negatively charged PPEs and their metal complexes. The array is sensitive towards the phenylhydantoin‐derivatives (PTH) of amino acids by modulation of the array‘s fluorescence intensity. In the first step we construct a sensor array investigating PTH‐amino acid standards (Figure 1 a) and optimize recognition/discrimination; in the second step we degrade an oligopeptide by N‐terminal Edman sequencing chemistry and identify the products with our array (Figure 1 b, c).

**Figure 1:**
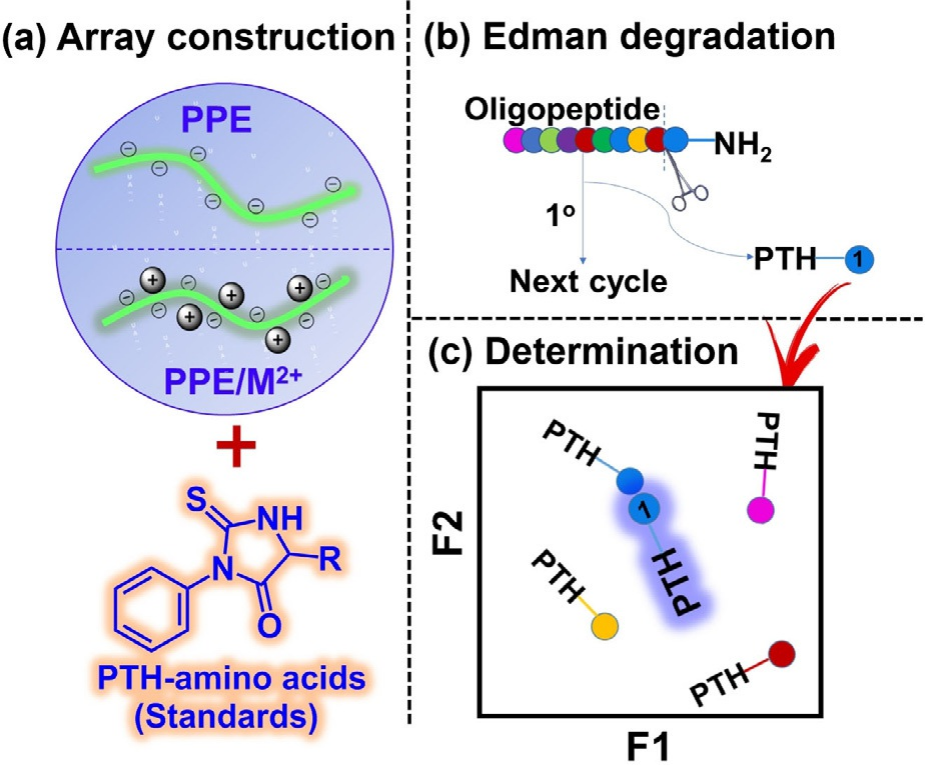
Schematic representation of the sensor array using PPEs. (a) An array involving PPEs and metal cations was constructed to identify PTH‐amino acid standards. (b) N‐terminal Edman degradation. (c) Determination of the amino acids from Edman degradation using an array.

PTH‐amino acids derived from the twenty natural amino acids possess diverse structural characteristics.^1^ Their detection and discrimination represents an attractive alternative in oligopeptide or protein sequencing.^2^ Chromatographic techniques coupled with mass spectrometry^3^ usually analyze PTH‐amino acids, but require specialized instrumentation.^4^

Array‐based sensing methods^5^ based on organic fluorophores,^6^ nanoparticles^7^ or quantum dots^8^ form a flexible alternative, and recognize different analytes or identify mixed samples; they display operational simplicity and often high sensitivity. Our group has focused on water‐soluble conjugated poly(*p*‐phenyleneethynylene)s (PPEs) to construct small sensor arrays to discriminate wines,^9^ explosives,^10^ sugars,^11^ bacteria,^12^ teas^13^ and other analytes.^14^ Here, we investigate the PPEs alone and with transition metal cations as adjuvants to discriminate 20 different PTH‐amino acids (Figure 2)—products of the Edman degradation of proteins/peptides.

**Figure 2:**
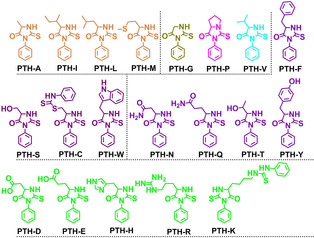
Structures of the investigated standard PTH‐amino acids.

In the initial study, seven PPEs, including the negatively charged **P1**–**P3**, neutral **P4**–**P5** and positively charged **P6**–**P7** were chosen as sensing elements (Figure 3 a and Figure S1). We treated **P2**, **P5** and **P6** (2 μm) with 12 randomly chosen PTH‐amino acids (1 mg mL^−1^) in four different solvents, including DMSO, DMSO/MeOH (1:1), DMSO/acetone (1:1), DMSO/H_2_O (1:1). PPEs with negative charges (**P2**) showed the strongest response to PTH‐amino acids in DMSO/H_2_O (1:1), followed by neutral **P5** and positively charged **P6** (Figure S2). After this cursory evaluation, we employed the negatively charged and neutral PPEs (**P1**–**P5**) in DMSO/H_2_O (1:1) for the discrimination experiments. However, most of the PTH‐amino acids group together quite tightly in the bottom right part of the LDA graph (Figure S3).

**Figure 3:**
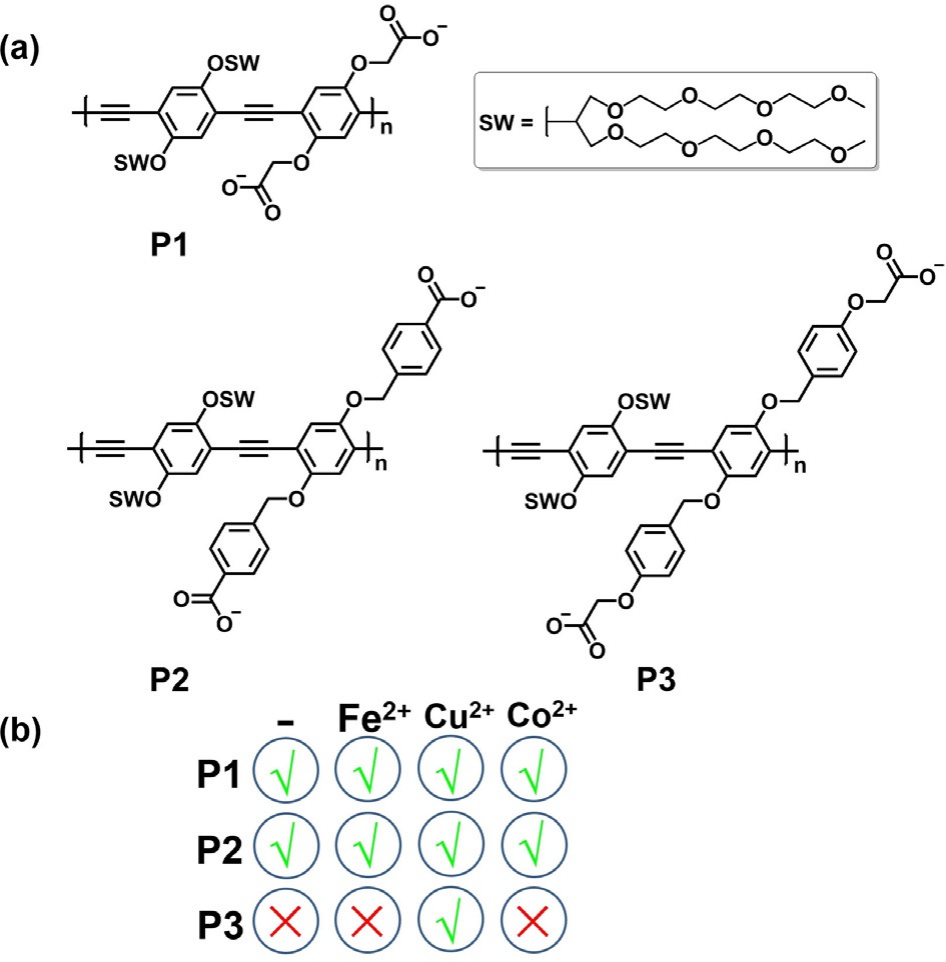
(a) Chemical structures of the poly(*p*‐phenyleneethynylene)s **P1**–**P3**. (b) Systematic screening of the successful array elements for sensing.

To construct a more discriminating array, we investigated the fluorescence response of negatively charged **P1**–**P3** towards PTH‐amino acids in the presence of metal cations (Fe^2+^, Cu^2+^, Co^2+^, Figure 3 b). **P1**–**P3** were first mixed with metal cations. After adding analytes we observed different levels of quenching as well as fluorescence turn‐on. In this study, about 10 μm Fe^2+^, 10 μm Cu^2+^ and 1 mm Co^2+^ were selected to obtain robust responses (Figure S4). The interaction of **P3** with PTH‐amino acid or metal ions was completed within 20 min at ambient temperature, while the **P3**‐Cu^2+^ complex showed slow response, achieving a plateau after 2 h (Figure S5). Accordingly, we selected an incubation time of 2 h at ambient temperature. Figure S6 shows the response patterns of the twelve‐element sensor array. Three negatively charged fluorescent **P1**–**P3** and their complexes with metal cations display binding interaction for a specific analyte, thus discriminating the PTH‐amino acids. Five measurements were performed with all PTH‐amino acids to provide a training matrix of 12 sensing elements × 20 PTH‐amino acids × 5 replicates. The resulting training data were analyzed and processed through LDA^15^ by the SYSTAT software. The first two factors allow to construct the two‐dimensional (2D) discrimination plot (Figure S7).

We performed principal component analysis (PCA, see Figure S9) to find the final nine elements that contribute most to the discrimination and used this optimized nine‐element tongue. Figure 4 shows the linear discriminant analysis (LDA) plot of all of the investigated PTH‐amino acids based on these nine elements. The jackknifed classification matrix with cross validation reveals 100 % accuracy. Furthermore, the PTH‐amino acid with identical molecular weight and similar structure, PTH‐I and PTH‐L (248 Da), are easily discriminated. The 20 PTH‐amino acids were discriminated; a simple nine‐element sensor array adequately identifies all of PTH‐amino acids. A well‐clustered three‐dimensional plot displaying the unique pattern is shown in Figure S11. To further validate the efficiency of the optimized sensing system, we tested 60 samples of PTH‐amino acids controlled at the same concentration of 1 mg mL^−1^ but with unknown identity, randomly chosen from the 20 PTH‐amino acids. The new cases were classified into groups, generated from the training matrix, based on the shortest Mahalanobis distance to the respective group.^16^ 53 of 60 unknown PTH‐amino acids were correctly identified, representing an accuracy of 88 %. These results confirmed that this sensor array works effectively in determination of PTH‐amino acids.

**Figure 4:**
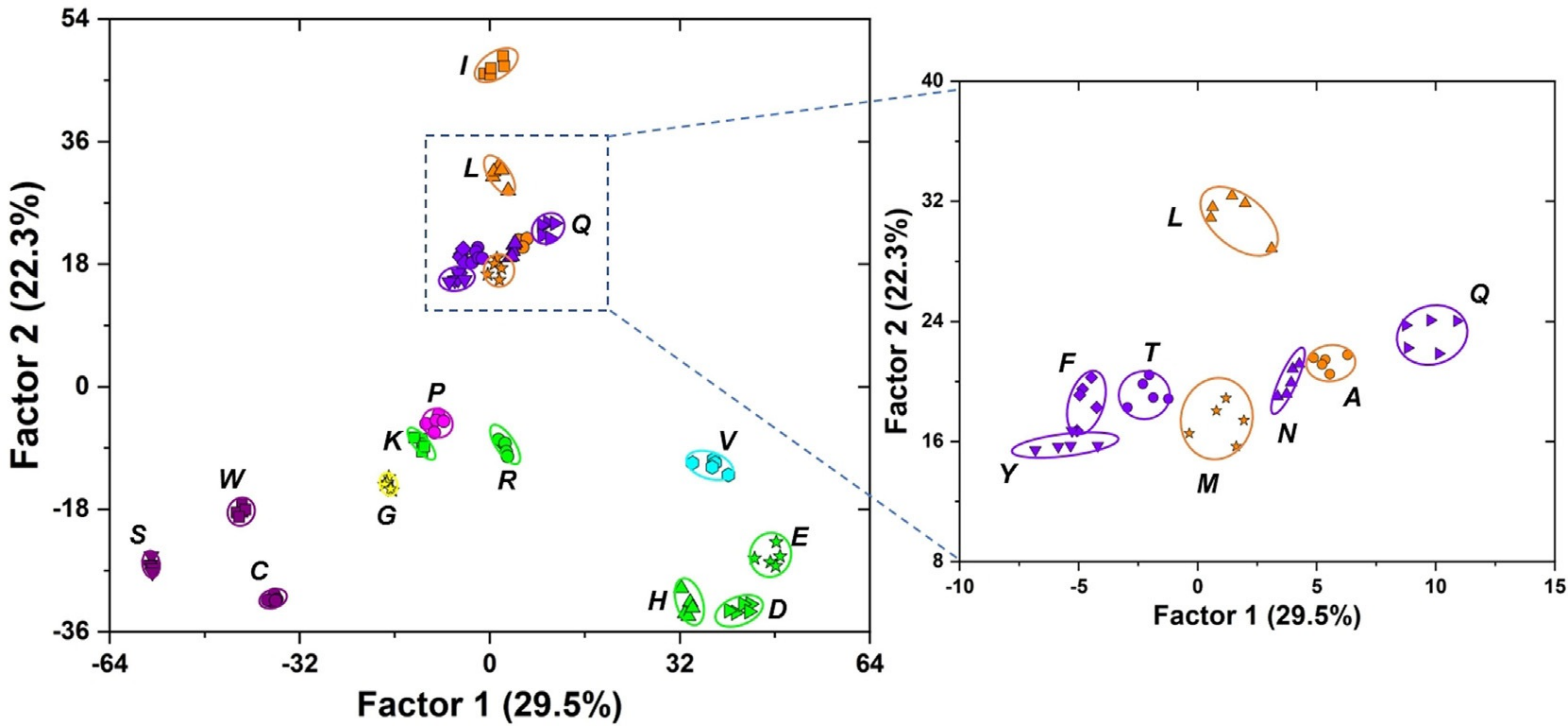
2D canonical score plot for the first two factors of fluorescence response patterns obtained by a nine‐element sensor array with 95 % confidence ellipses. ′PTH′ was omitted for clarity.

Discrimination is based on two interactions (Figure 5), the direct interaction between the negatively charged **P1**–**P3** and the PTH‐amino acids and the competitive interaction among polymers, analytes and metal cations. The direct interaction between PPE and the PTH‐amino acid could lead to minor changes or decreases of fluorescence intensity, which may be caused by the π–π stacking interaction between the PPEs and quenchers or the hydrophobic interactions.10a, 14 Metal ions are not only fluorescence quenchers, but also bind to a wide range of biomolecules with chelating groups like −C=O and −NH.^17^ The addition of Fe^2+^, Cu^2+^ or Co^2+^ results in a decrease in fluorescence intensity of **P1–P3** (Figure S12), probably because of the formation of electrostatic complexes or coordination of metal ions to the branched oligoethylene glycol moieties on PPEs.^18^ The metal‐quenched fluorescence was modulated upon the addition of PTH‐amino acids. PPEs was released and the fluorescence turn‐on is caused by displacement; The observed fluorescence turn‐off is probably because of the further binding of PTH‐amino acids to the PPE‐metal complexes, thus forming ternary complexes (Figure S13). These results are consistent with a displacement model.^19^

**Figure 5:**
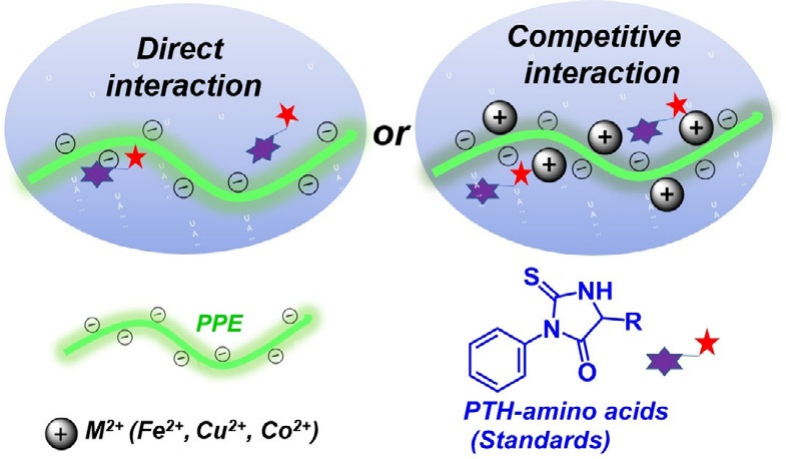
Schematic illustration of two ways of interaction in constructing a sensor array.

Edman degradation is a standard cyclic three‐step method for peptide sequencing (Figure 6), in which the N‐terminal amino acid is cleaved off and identified.^2^ To explore the application of our sensor array, Edman degradation was performed on the peptide, NH_2_‐Met‐Ala‐Ser‐OH (Figure 7 a). Due to the limit to manual degradation, two cycles were performed by the three‐stage modified Edman procedure (see the Supporting Information), with a yield of 80 % and 62 %, respectively. The array response to each degradation residue (1 mg mL^−1^) was compared to that of the classification data from the nine‐element sensor array and the two residues were correctly identified according to their placement in 2D‐LDA plot in Figure 7 b. The residue from the first cycle **C1** ends up in the PTH‐M zone, while **C2** (residue from cycle 2°) was congruent with PTH‐A, as expected from the oligopeptide structure. GC‐MS analyses also identify the degradation residues correctly (Table S5). Our methodology based on the sensor array could provide a complementary way to determinate PTH‐amino acids.

**Figure 6:**
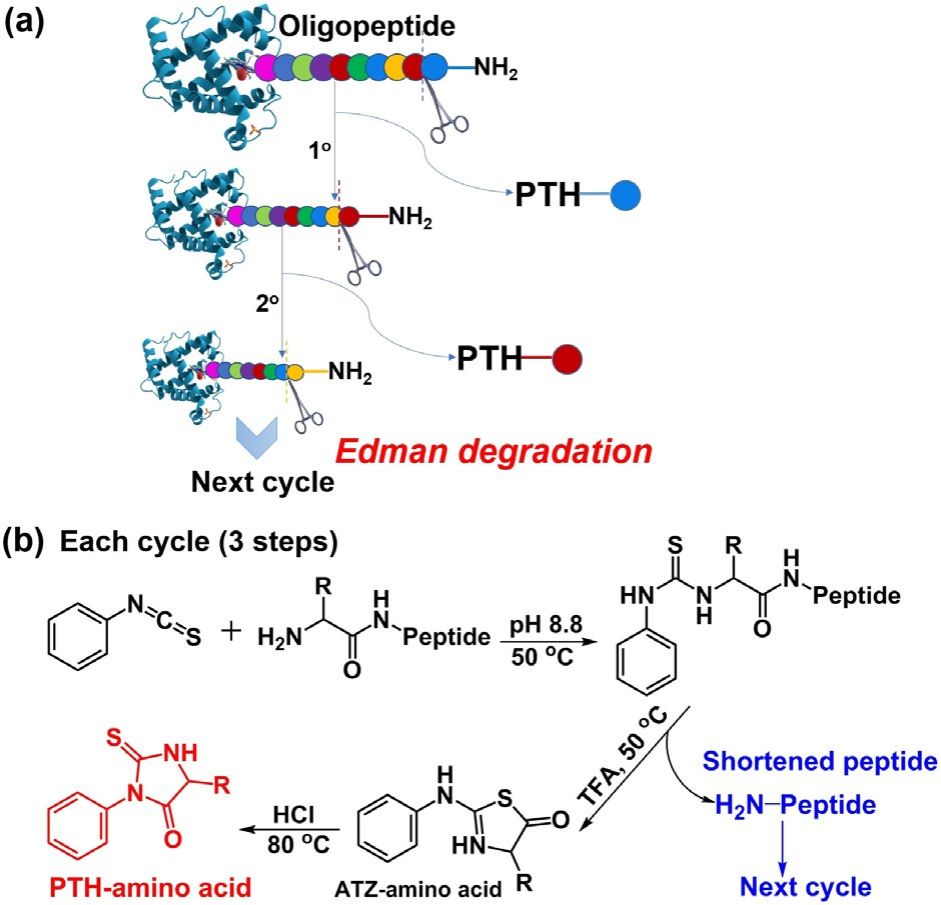
(a) N‐terminal Edman sequencing chemistry and (b) detailed procedure for each cycle.

**Figure 7:**
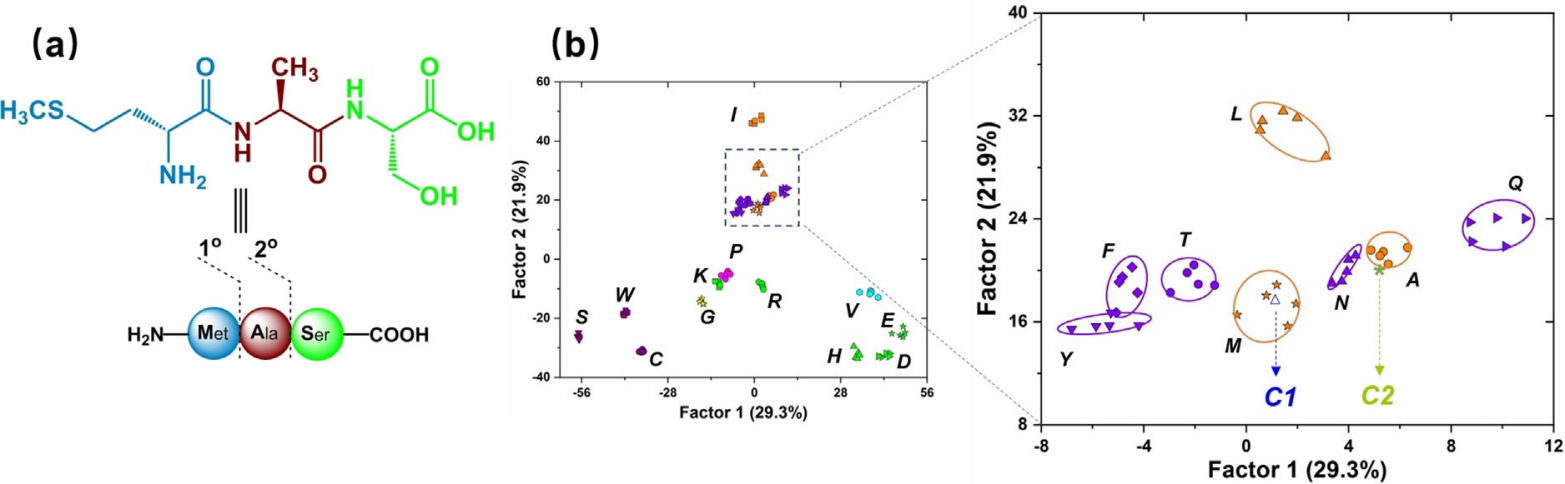
(a) The oligopeptide used for degradation. (b) Degradation residues (**C1** and **C2**, the average of three measurements) were clustered with the established reference PTH‐amino acids via LDA. ′PTH′ was omitted for clarity.

In conclusion, we developed a nine‐element sensor array using three anionic PPEs, together with their metal complexes. These identify twenty different PTH‐amino acids. This sensor array may provide a potential and powerful tool to determinate the primary structure of oligopeptide and proteins after Edman degradation.

## Conflict of interest

The authors declare no conflict of interest.

## Supporting information

As a service to our authors and readers, this journal provides supporting information supplied by the authors. Such materials are peer reviewed and may be re‐organized for online delivery, but are not copy‐edited or typeset. Technical support issues arising from supporting information (other than missing files) should be addressed to the authors.

Supplementary
