# Supplementary material for: Sensor Array Based Determination of Edman Degradated Amino Acids Using Poly(p‐phenyleneethynylene)s
Source: Chemistry. 2020 May 29;26(35):7779–82. doi: 10.1002/chem.202001262 (PMC7383564; doi:10.1002/chem.202001262)
Supplement: Supplementary file 1 — Supplementary [file CHEM-26-7779-s001.pdf]

// Chemistry–A European Journal

Supporting Information

Sensor Array Based Determination of Edman Degradated Amino Acids Using Poly(*p*-phenyleneethynylene)s

Hao Zhang,[a] Benhua Wang,[b] Kai Seehafer,[a] and Uwe H. F. Bunz*[a, c]

Contents

1. General information 2

2. Figures and tables 3

3. Detailed procedure for Edman degradation 17

4. References 18

1. General information

1.1 Materials. Chemicals and solvents were either purchased from the chemical store at the Organisch-Chemisches Institut of the University of Heidelberg or from commercial laboratory suppliers. Standard PTH-amino acids were purchased from TCI or Santa Cruz Biotechnology. Inc. Metal salts were used as $Fe(ClO_4)_2{\bullet}xH_2O$, $Cu(ClO_4)_2{\bullet}6H_2O$, and $Co(ClO_4)_2{\bullet}6H_2O$. Reagents were used without further purification unless otherwise noted. The synthesis of PPEs **P1-P7** were reported previously.[1]

1.2 Method for Fluorescence response pattern. Fluorescence intensity (I_0 or I) was recorded on a CLARIOstar (firmware version 1.13) Platereader from BMG Labtech using the corresponding software (software version 5.20 R5). Data were analyzed with CLARIOstar MARS Data Analysis Software (software version 3.10 R5) from BMG Labtech. I_0 and I are the fluorescence intensity of the solution in the absence and presence of the PTH-amino acids, respectively. To ensure solubility, stock solutions of all the analyte PTH-amino acid were prepared in DMSO and diluted using water (2 mg/mL). The stock solutions for metal ions (10 mM) and PPEs (**P1**, **P3**: 1 mM; **P2**, 0.4 mM) were prepared in water and diluted using DMSO as needed in the sensing experiments. 150 μL of polymers or the polymer- metal ions solution in DMSO/H_2O (1:1) was loaded into a well on a 96-well plate (300 μL microplate) first. Subsequently, 150 μL PTH-amino acids solution were added to each well. After incubation for 2 h at room temperature, the fluorescence intensity values were recorded on the microplate reader with an excitation at 410 nm for **P1**, 430 nm for **P2** and **P3**.

1.3 Linear discriminant analysis (LDA) was carried out using classical linear discriminant analysis in SYSTAT (version 13.0). In LDA, all variables were used in the model (complete mode) and the tolerance was set as 0.001. The fluorescence response patterns were transformed to canonical patterns. The Mahalanobis distances of each individual pattern to the centroid of each group in a multidimensional space were calculated and the assignment of the case was based on the shortest Mahalanobis distance.

1.4 Principal component analysis (PCA) was carried out in excel using xlstat by reducing the redundancy in the dimensionality of the data. All data points for the analytes were taken and generated a set of orthogonal eigenvectors (principal components, PCs) for maximum variance.

2. Figures and tables

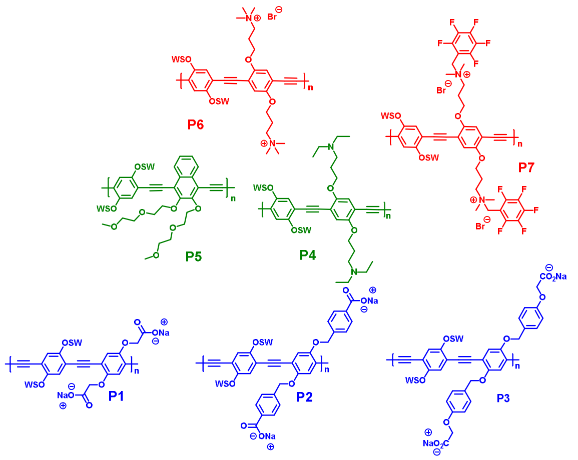

Figure S1. Structures of PPEs **P1-P7** used for initial screening.

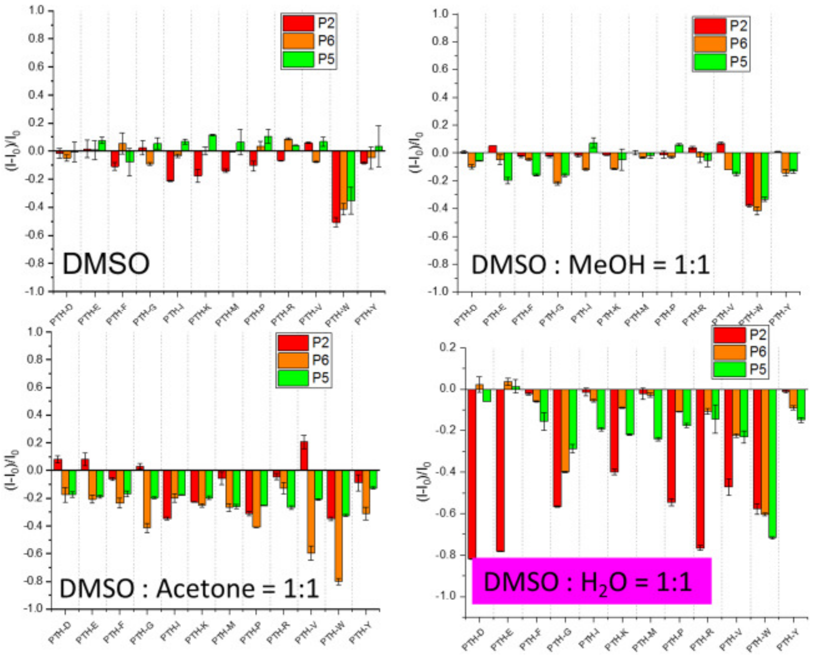

Figure S2. Fluorescence response generated by **P2**, **P5** and **P6** (2 μM) with 12 randomly chosen PTH-amino acids (1 mg/mL) in four different solvents, including DMSO, DMSO/MeOH (1:1), DMSO/acetone (1:1), DMSO/H_2O (1:1).

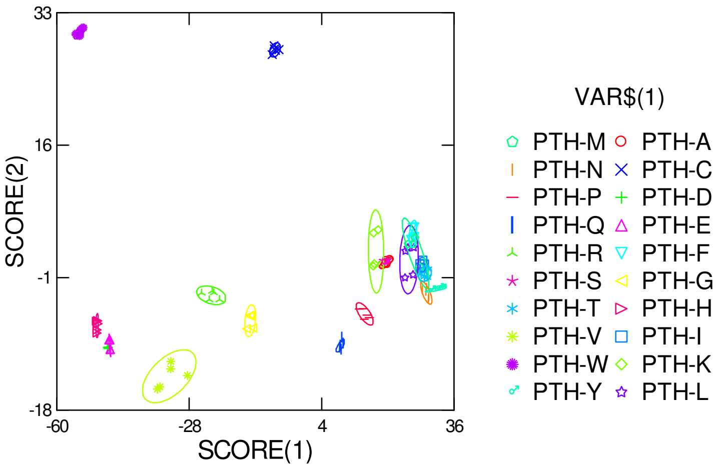

Figure S3. 2D canonical score plot of fluorescence response patterns obtained by the neutral and negatively charge PPEs **P1-P5** (2 μM) against PTH-amino acids (1 mg/mL).

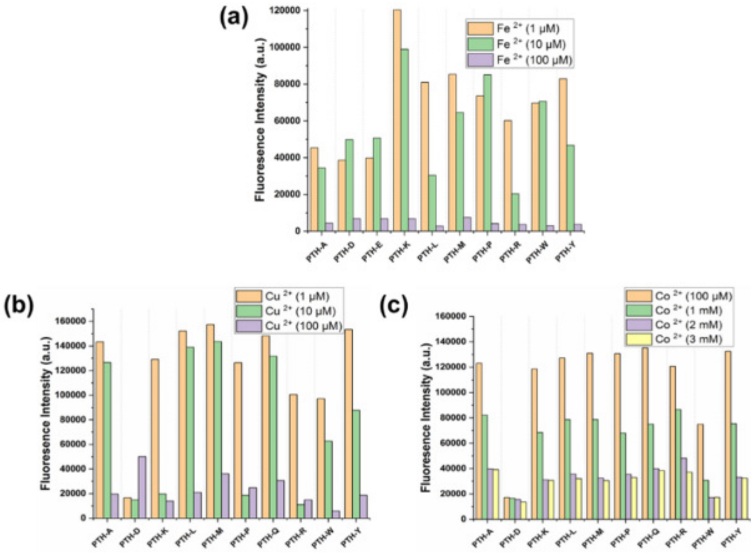

Figure S4. Fluorescence intensity of **P3** (2 μM) with different metal ions at different concentration in the presence of randomly selected analytes.

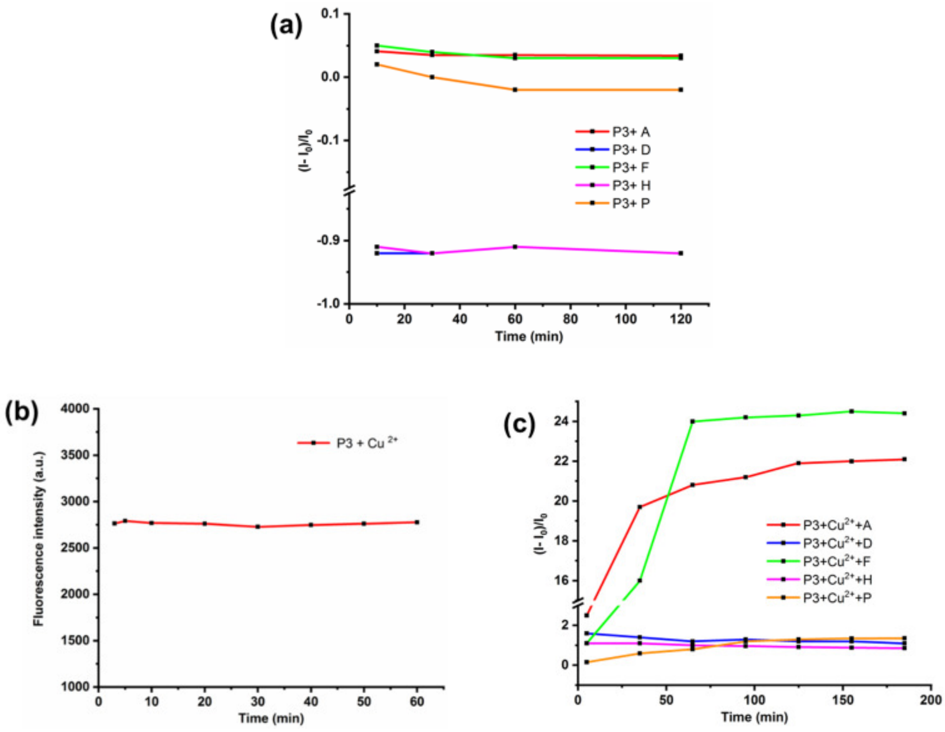

Figure S5. Interaction time study of among **P3** (2 μM), PTH-amino acids (1 mg/mL) and Cu^{2+} (10 μM).

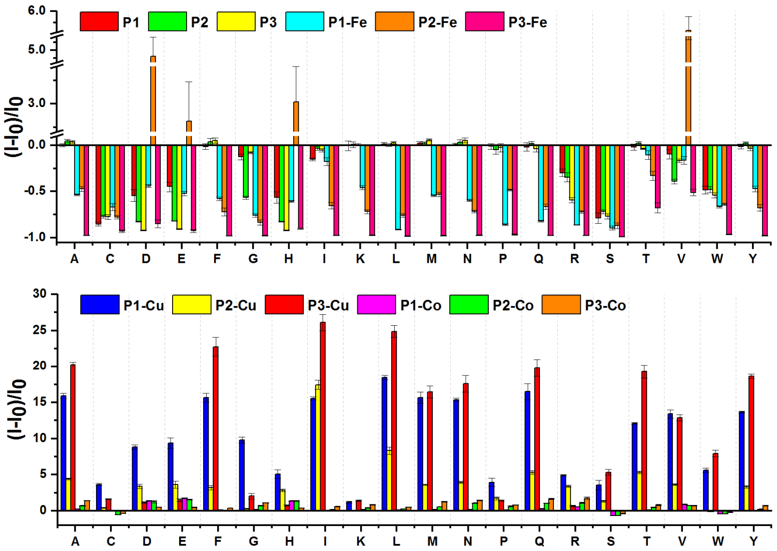

Figure S6. Fluorescence response pattern ($(I - I_0)/I_0$) obtained by **P1-P3** (2 μM) and their metal-complexes (Fe^{2+}, Cu^{2+}: 10 μM; Co^{2+}: 1 mM) treated with PTH-amino acids (1 mg/mL) in DMSO/H_2O (1:1) after incubation for 2 h at room temperature. Each value is the average of five independent measurements and each error bar shows the standard error of these measurements. 'PTH' was omitted for clarity.

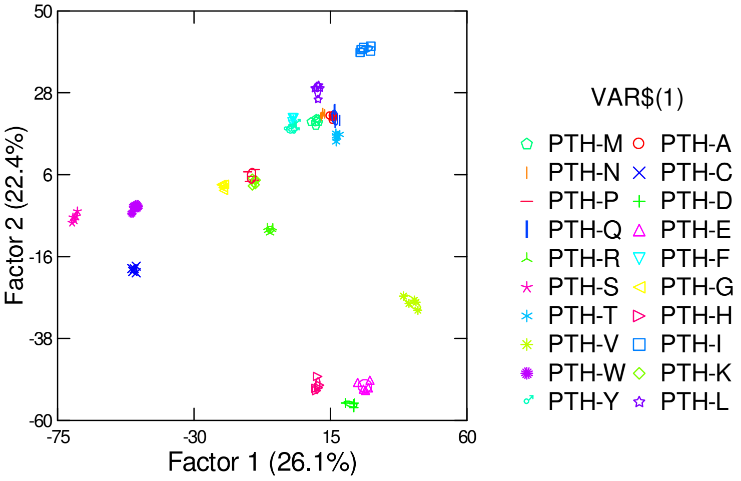

Figure S7. 2D canonical score plot for the first two factors of fluorescence response patterns obtained by a twelve-element sensor array. Each point represents the response pattern for a single PTH-amino acid in the array.

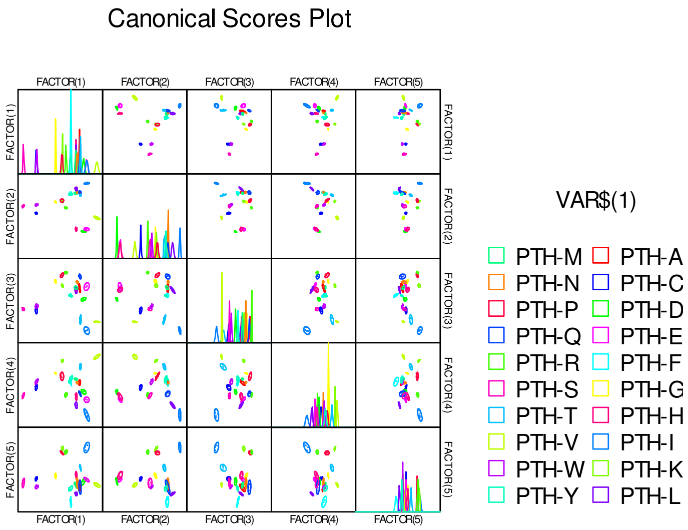

Figure S8. Correlations of canonical fluorescence response patterns from an array of twelve elements against twenty PTH-amino acids.

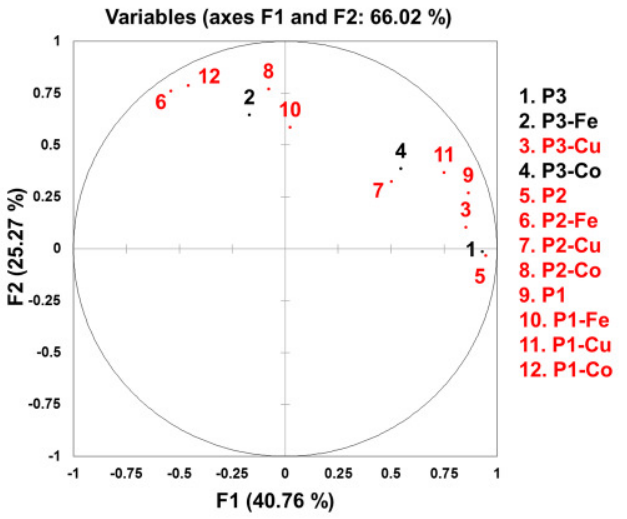

Figure S9. Loading plot of the principal component analysis, identifying the contribution of each element to an axis. The finally selected nine elements are labeled in red.

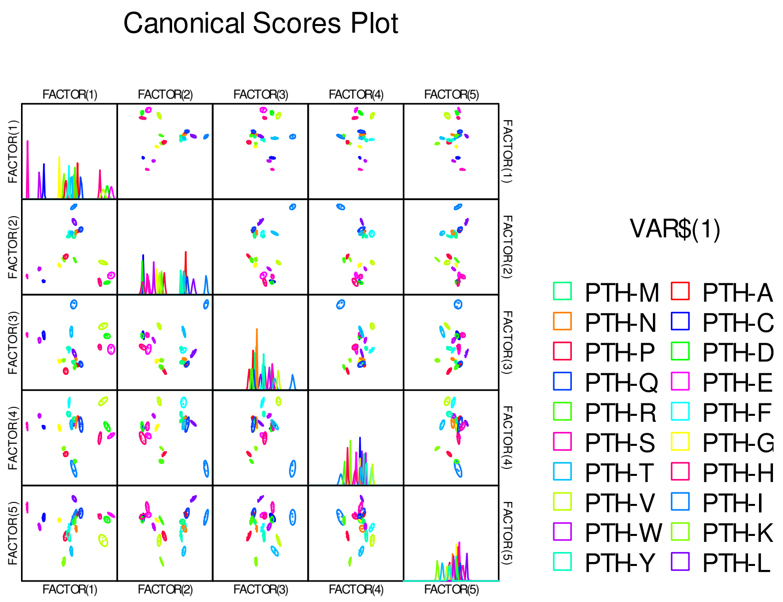

Figure S10. Correlations of canonical fluorescence response patterns from an array of nine elements against twenty PTH-amino acids.

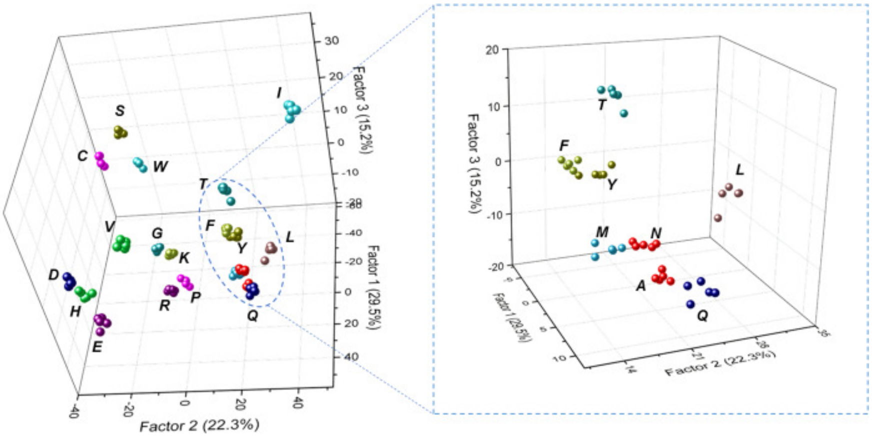

Figure S11. 3D canonical score plot for the first three factors of fluorescence response patterns obtained by a nine-element sensor array. Each point represents the response pattern for a single PTH-amino acid in the array. 'PTH' was omitted for clarity.

Table S1. Training matrix of fluorescence response pattern from an array of twelve elements against twenty PTH-amino acids.

Analyte	Fluorescence response pattern											
PTH-amino acids	P1	P2	P3	P1-Fe^{2+}	P2-Fe^{2+}	P3-Fe^{2+}	P1-Cu^{2+}	P2-Cu^{2+}	P3-Cu^{2+}	P1-Co^{2+}	P2-Co^{2+}	P3-Co^{2+}
PTH-A	-0.022	0.066	0.050	-0.497	-0.485	-0.975	15.553	4.455	20.110	0.215	0.736	1.369
PTH-A	0.001	0.060	0.054	-0.586	-0.451	-0.979	16.029	4.235	19.869	0.209	0.719	1.415
PTH-A	0.021	0.049	0.035	-0.494	-0.452	-0.969	15.584	4.539	20.745	0.217	0.672	1.444
PTH-A	0.009	0.038	0.027	-0.586	-0.475	-0.973	16.460	4.495	20.576	0.254	0.690	1.408
PTH-A	0.000	0.019	0.027	-0.495	-0.478	-0.974	15.857	4.390	19.958	0.258	0.709	1.409
PTH-C	-0.817	-0.742	-0.746	-0.698	-0.798	-0.916	3.454	0.380	1.610	0.032	-0.522	-0.351
PTH-C	-0.870	-0.775	-0.751	-0.699	-0.782	-0.915	3.559	0.400	1.570	0.046	-0.540	-0.340
PTH-C	-0.860	-0.773	-0.782	-0.648	-0.754	-0.936	3.632	0.430	1.720	0.027	-0.595	-0.354
PTH-C	-0.860	-0.785	-0.793	-0.691	-0.794	-0.947	3.862	0.470	1.660	0.066	-0.513	-0.429
PTH-C	-0.850	-0.782	-0.805	-0.617	-0.767	-0.930	3.648	0.420	1.660	0.074	-0.569	-0.334
PTH-D	-0.472	-0.819	-0.915	-0.417	4.867	-0.898	8.767	3.471	1.299	1.473	1.116	0.468
PTH-D	-0.482	-0.827	-0.917	-0.458	5.269	-0.887	8.862	3.503	1.148	1.348	1.091	0.447
PTH-D	-0.596	-0.831	-0.920	-0.458	4.925	-0.805	9.012	3.638	1.121	1.361	1.417	0.463
PTH-D	-0.588	-0.827	-0.924	-0.439	4.957	-0.826	8.388	3.271	1.001	1.395	1.463	0.504
PTH-D	-0.588	-0.829	-0.918	-0.439	4.596	-0.826	9.092	2.858	1.069	1.383	1.212	0.447
PTH-E	-0.388	-0.812	-0.902	-0.500	2.723	-0.941	10.212	3.931	1.617	1.748	1.549	0.497
PTH-E	-0.416	-0.821	-0.904	-0.500	3.141	-0.944	9.768	3.817	1.435	1.726	1.578	0.434
PTH-E	-0.432	-0.821	-0.906	-0.535	3.356	-0.911	9.535	3.864	1.151	1.646	1.586	0.376
PTH-E	-0.505	-0.818	-0.905	-0.533	2.375	-0.917	8.555	2.820	1.697	1.834	1.593	0.584
PTH-E	-0.505	-0.817	-0.908	-0.551	2.487	-0.917	8.696	3.660	1.571	1.719	1.432	0.471
PTH-F	-0.007	0.075	0.058	-0.590	-0.752	-0.980	15.220	3.345	22.585	0.077	-0.014	0.336

PTH-F	0.017	0.068	0.062	-0.599	-0.663	-0.983	14.995	3.304	22.853	0.069	-0.011	0.361
PTH-F	-0.065	0.037	0.030	-0.588	-0.711	-0.978	16.326	3.528	24.475	0.086	-0.014	0.330
PTH-F	-0.011	0.027	0.020	-0.562	-0.706	-0.979	15.549	3.006	23.011	0.108	0.064	0.345
PTH-F	0.000	-0.012	0.092	-0.561	-0.777	-0.978	16.260	2.826	20.793	0.124	0.009	0.376
PTH-G	-0.122	-0.546	-0.081	-0.740	-0.857	-0.975	10.242	0.277	2.540	0.145	0.648	1.071
PTH-G	-0.128	-0.575	-0.067	-0.738	-0.871	-0.981	10.247	0.169	2.188	0.137	0.756	1.058
PTH-G	-0.101	-0.561	-0.076	-0.775	-0.812	-0.978	9.581	0.284	2.027	0.129	0.685	1.150
PTH-G	-0.174	-0.565	-0.094	-0.763	-0.809	-0.977	9.328	0.287	1.970	0.183	0.671	1.147
PTH-G	-0.100	-0.589	-0.072	-0.781	-0.818	-0.975	9.651	0.336	1.441	0.165	0.755	1.140
PTH-H	-0.509	-0.819	-0.916	-0.604	3.145	-0.896	5.914	2.607	0.793	1.364	1.363	0.386
PTH-H	-0.514	-0.821	-0.920	-0.608	3.367	-0.896	4.586	2.708	0.578	1.330	1.359	0.396
PTH-H	-0.626	-0.829	-0.915	-0.615	3.188	-0.906	4.811	2.816	0.778	1.409	1.199	0.330
PTH-H	-0.633	-0.825	-0.923	-0.591	2.384	-0.906	5.507	3.142	0.802	1.398	1.449	0.368
PTH-H	-0.550	-0.833	-0.923	-0.617	3.028	-0.910	4.573	2.841	0.730	1.402	1.445	0.371
PTH-I	-0.157	-0.015	-0.048	-0.110	-0.654	-0.971	15.606	16.746	25.603	-0.034	0.184	0.566
PTH-I	-0.141	-0.007	-0.084	-0.213	-0.623	-0.978	15.420	17.029	24.629	-0.017	0.184	0.617
PTH-I	-0.153	-0.047	-0.030	-0.157	-0.629	-0.976	15.838	17.193	27.284	0.006	0.163	0.547
PTH-I	-0.174	-0.028	-0.050	-0.203	-0.711	-0.977	15.277	18.194	27.093	0.058	0.168	0.579
PTH-I	-0.140	-0.061	-0.050	-0.188	-0.652	-0.974	15.577	17.973	25.849	0.084	0.151	0.560
PTH-K	-0.022	0.049	0.012	-0.444	-0.729	-0.972	1.194	0.041	1.216	0.147	0.329	0.842
PTH-K	-0.033	0.018	0.024	-0.462	-0.686	-0.977	1.126	0.015	1.390	0.136	0.459	0.904
PTH-K	0.081	0.012	-0.002	-0.451	-0.710	-0.972	1.177	0.101	1.446	0.103	0.427	0.841
PTH-K	-0.051	-0.013	-0.007	-0.495	-0.737	-0.972	1.131	-0.063	1.435	0.185	0.362	0.860
PTH-K	0.000	-0.028	0.001	-0.452	-0.729	-0.972	1.351	0.151	1.501	0.194	0.384	0.798
PTH-L	0.001	0.008	0.041	-0.911	-0.766	-0.982	18.637	8.237	24.231	0.069	0.253	0.476
PTH-L	0.005	0.023	0.045	-0.911	-0.748	-0.984	18.765	8.561	25.180	0.075	0.255	0.468
PTH-L	0.011	-0.004	0.011	-0.913	-0.758	-0.979	18.540	8.618	25.192	0.082	0.203	0.527
PTH-L	0.049	-0.016	0.024	-0.914	-0.734	-0.983	18.003	8.770	25.908	0.114	0.301	0.547
PTH-L	0.000	0.005	0.024	-0.913	-0.790	-0.983	18.238	7.481	23.822	0.171	0.292	0.501
PTH-M	0.048	0.049	0.049	-0.532	-0.525	-0.979	15.866	3.639	17.389	0.155	0.539	1.270
PTH-M	0.028	0.017	0.048	-0.551	-0.513	-0.981	15.009	3.602	16.317	0.140	0.522	1.269
PTH-M	0.031	0.018	0.060	-0.556	-0.523	-0.975	16.881	3.672	15.608	0.141	0.551	1.280
PTH-M	0.012	0.019	0.080	-0.536	-0.532	-0.978	15.380	3.708	17.299	0.206	0.518	1.177
PTH-M	0.000	0.007	0.057	-0.554	-0.578	-0.978	15.083	3.469	15.682	0.217	0.529	1.314
PTH-N	0.028	0.057	0.041	-0.602	-0.741	-0.972	15.171	3.804	18.812	0.138	1.055	1.416
PTH-N	0.003	0.043	0.056	-0.591	-0.709	-0.977	15.157	3.801	18.007	0.145	1.051	1.325
PTH-N	0.023	0.059	0.038	-0.592	-0.719	-0.972	15.602	4.040	18.131	0.148	0.985	1.419
PTH-N	-0.012	0.011	0.030	-0.587	-0.709	-0.971	15.325	4.099	15.860	0.132	1.105	1.535
PTH-N	0.000	-0.004	0.094	-0.618	-0.704	-0.971	15.627	3.986	17.178	0.152	1.081	1.541
PTH-P	0.018	0.012	0.032	-0.855	-0.478	-0.969	4.318	1.391	1.350	0.069	0.611	0.803
PTH-P	0.013	-0.018	0.011	-0.867	-0.504	-0.972	3.357	1.564	1.316	0.060	0.504	0.766
PTH-P	-0.027	-0.054	-0.035	-0.849	-0.481	-0.961	4.417	1.765	1.456	0.089	0.636	0.765
PTH-P	-0.056	-0.093	-0.060	-0.866	-0.482	-0.961	3.282	1.937	1.473	0.126	0.609	0.825
PTH-P	0.000	-0.091	-0.074	-0.851	-0.489	-0.961	4.319	1.908	1.539	0.123	0.776	0.835
PTH-Q	0.013	0.052	-0.015	-0.810	-0.633	-0.975	16.467	5.214	20.329	0.260	1.026	1.522

PTH-Q	-0.099	0.013	0.001	-0.829	-0.669	-0.977	17.497	5.176	21.086	0.235	1.054	1.795
PTH-Q	0.001	0.029	-0.026	-0.818	-0.645	-0.971	14.640	5.401	20.169	0.275	1.018	1.642
PTH-Q	-0.004	0.002	-0.080	-0.809	-0.674	-0.972	17.356	5.659	19.442	0.318	1.008	1.586
PTH-Q	0.000	0.000	-0.063	-0.825	-0.703	-0.972	16.448	5.134	17.946	0.303	1.110	1.668
PTH-R	-0.239	-0.333	-0.589	-0.860	-0.739	-0.975	5.023	3.227	0.725	0.532	1.142	1.490
PTH-R	-0.274	-0.294	-0.619	-0.861	-0.709	-0.977	4.866	3.321	0.684	0.491	1.087	1.691
PTH-R	-0.337	-0.302	-0.599	-0.866	-0.707	-0.972	4.837	3.514	0.722	0.558	0.964	1.869
PTH-R	-0.331	-0.383	-0.617	-0.861	-0.724	-0.975	4.978	3.587	0.442	0.577	1.152	1.771
PTH-R	-0.300	-0.406	-0.550	-0.863	-0.733	-0.975	4.803	3.355	0.724	0.596	1.205	1.872
PTH-S	-0.723	-0.702	-0.730	-0.887	-0.908	-0.990	3.738	1.291	5.892	-0.669	-0.651	-0.352
PTH-S	-0.740	-0.702	-0.758	-0.866	-0.839	-0.991	2.624	1.336	5.396	-0.662	-0.625	-0.350
PTH-S	-0.813	-0.735	-0.767	-0.909	-0.874	-0.990	4.373	1.293	5.382	-0.659	-0.709	-0.434
PTH-S	-0.856	-0.713	-0.795	-0.891	-0.838	-0.990	3.215	1.481	5.204	-0.646	-0.684	-0.350
PTH-S	-0.810	-0.742	-0.796	-0.917	-0.881	-0.990	3.850	1.122	4.796	-0.635	-0.655	-0.407
PTH-T	0.026	0.046	-0.026	-0.187	-0.369	-0.620	12.237	5.122	20.647	0.071	0.474	0.839
PTH-T	-0.056	0.047	-0.041	-0.094	-0.358	-0.696	11.962	5.215	19.704	0.061	0.521	0.900
PTH-T	-0.038	0.007	-0.035	-0.105	-0.271	-0.624	12.192	5.275	18.772	0.084	0.373	0.630
PTH-T	-0.033	-0.001	-0.040	-0.079	-0.362	-0.726	12.115	5.625	18.342	0.123	0.419	0.823
PTH-T	0.000	0.006	-0.044	-0.062	-0.278	-0.726	11.905	5.336	18.893	0.116	0.512	0.867
PTH-V	-0.119	-0.386	-0.170	-0.151	5.992	-0.513	13.718	3.508	12.559	0.889	0.778	0.798
PTH-V	-0.164	-0.351	-0.158	-0.143	5.358	-0.503	13.963	3.505	12.304	0.869	0.757	0.719
PTH-V	-0.033	-0.386	-0.143	-0.208	5.721	-0.473	13.731	3.738	13.361	0.857	0.708	0.744
PTH-V	-0.070	-0.422	-0.158	-0.198	5.064	-0.506	13.004	3.730	13.184	0.855	0.708	0.666
PTH-V	-0.100	-0.399	-0.193	-0.103	5.336	-0.568	12.569	3.670	12.961	0.915	0.757	0.704
PTH-W	-0.452	-0.483	-0.534	-0.649	-0.660	-0.960	5.125	-0.147	8.582	-0.392	-0.427	-0.185
PTH-W	-0.433	-0.439	-0.539	-0.678	-0.643	-0.968	5.735	-0.092	7.347	-0.436	-0.441	-0.186
PTH-W	-0.507	-0.491	-0.532	-0.678	-0.634	-0.963	5.808	0.010	7.742	-0.438	-0.415	-0.213
PTH-W	-0.540	-0.470	-0.520	-0.659	-0.632	-0.963	5.750	0.041	8.103	-0.400	-0.430	-0.233
PTH-W	-0.500	-0.524	-0.594	-0.669	-0.628	-0.963	5.601	0.081	7.921	-0.409	-0.384	-0.269
PTH-Y	-0.004	0.036	-0.023	-0.475	-0.694	-0.977	13.637	3.076	18.551	0.061	0.198	0.720
PTH-Y	0.014	0.041	-0.006	-0.431	-0.669	-0.980	13.719	3.348	18.694	0.059	0.240	0.769
PTH-Y	-0.023	0.012	-0.047	-0.476	-0.631	-0.977	13.719	3.287	19.149	0.065	0.267	0.668
PTH-Y	-0.052	0.004	-0.063	-0.518	-0.728	-0.977	13.769	3.512	18.475	0.054	0.154	0.706
PTH-Y	0.000	0.025	-0.035	-0.470	-0.664	-0.977	13.461	3.376	18.288	0.109	0.233	0.600

Table S2. LDA was carried out as described above resulting in the final nine factors of the canonical scores and group generation.

Analyte	Resuls LDA (nine scores)									
PTH-amino acids	Group	(1)	(2)	(3)	(4)	(5)	6)	(7)	(8)	(9)
PTH-A	1	5.62	21.84	-4.48	6.87	-1.02	-1.36	-0.05	2.92	-1.11
PTH-A	1	5.47	21.16	-6.57	7.48	1.59	-0.16	-0.02	1.87	-1.10
PTH-A	1	5.15	21.97	-3.67	7.71	-0.73	-1.61	0.14	1.93	0.27
PTH-A	1	6.52	21.84	-5.71	7.92	2.67	-0.87	0.62	1.23	-0.50
PTH-A	1	5.93	20.83	-3.97	7.71	-0.15	-2.56	0.00	1.61	-0.37
PTH-C	2	-35.98	-30.59	9.84	-0.31	2.14	-2.99	5.66	-0.70	-2.19
PTH-C	2	-36.25	-31.66	11.05	-0.07	2.92	-3.32	5.91	-0.31	-2.64
PTH-C	2	-37.00	-31.30	12.57	0.54	1.51	-3.93	5.81	-0.76	-2.70
PTH-C	2	-35.51	-31.30	11.14	0.08	3.18	-3.95	5.67	-0.56	-2.54
PTH-C	2	-35.63	-31.30	12.74	0.67	0.89	-4.98	6.00	-0.95	-2.45
PTH-D	3	44.27	-31.59	10.49	0.70	0.31	1.94	0.56	-2.56	-0.94
PTH-D	3	41.78	-32.91	11.75	1.13	1.12	4.72	-1.59	-2.29	-1.33
PTH-D	3	42.99	-31.69	10.31	-1.00	2.03	3.14	-3.41	2.12	-2.87
PTH-D	3	44.01	-32.77	9.51	-0.46	0.86	2.87	-3.13	2.84	-2.16
PTH-D	3	40.62	-32.61	9.85	1.77	1.36	1.35	-0.80	0.36	-2.95
PTH-E	4	48.31	-22.81	-0.34	-4.66	5.57	-7.85	2.70	-2.87	0.52
PTH-E	4	48.99	-24.87	0.72	-4.20	5.09	-6.01	1.84	-1.78	0.45
PTH-E	4	47.35	-25.81	1.05	-4.70	5.55	-4.01	0.51	-1.16	0.18
PTH-E	4	48.21	-26.41	-2.91	-3.42	5.66	-8.46	5.75	0.71	1.02
PTH-E	4	44.66	-25.29	-0.16	-5.56	6.12	-6.61	5.10	-0.80	0.38
PTH-F	5	-5.09	19.57	-2.31	14.01	1.00	0.83	6.30	-0.97	0.87
PTH-F	5	-5.22	19.14	-2.31	14.40	1.13	1.25	6.00	-0.98	1.85
PTH-F	5	-4.71	20.38	0.14	16.47	3.24	0.13	6.29	0.18	0.88
PTH-F	5	-4.46	18.40	-1.63	16.17	1.37	-0.81	5.37	-0.34	1.70
PTH-F	5	-5.29	16.85	-2.02	15.62	1.73	-2.25	4.89	-3.21	0.01
PTH-G	6	-17.21	-13.61	-9.30	2.17	4.45	-7.75	-7.33	-5.68	0.70
PTH-G	6	-17.47	-14.38	-9.56	2.06	4.77	-8.50	-8.84	-4.95	0.94
PTH-G	6	-17.91	-14.78	-10.15	1.02	4.88	-6.99	-8.02	-5.64	1.72
PTH-G	6	-16.59	-15.59	-9.41	0.76	4.71	-7.05	-6.57	-4.65	0.82
PTH-G	6	-16.91	-15.42	-10.57	0.30	5.48	-7.68	-8.59	-5.83	1.79
PTH-H	7	33.87	-31.78	1.15	-4.17	3.74	-0.57	0.58	2.15	2.41
PTH-H	7	33.02	-33.43	1.91	-5.47	2.66	1.19	0.26	3.11	3.52
PTH-H	7	33.64	-33.82	3.33	-5.38	3.74	0.79	3.82	3.08	1.70
PTH-H	7	32.40	-29.75	1.13	-7.90	4.25	-3.30	1.87	4.69	1.17
PTH-H	7	34.28	-32.65	0.85	-6.63	3.66	-0.45	1.14	4.06	3.54
PTH-I	8	-0.63	47.03	30.68	-23.91	-4.35	-1.92	-2.04	-0.75	-0.99
PTH-I	8	0.25	46.68	28.24	-26.48	-2.07	0.17	-1.53	-1.85	-1.20
PTH-I	8	0.38	47.77	31.59	-23.44	-1.62	-1.57	-1.22	-0.63	0.80
PTH-I	8	2.38	49.42	31.27	-27.97	-0.60	-0.24	0.21	-0.66	0.65
PTH-I	8	2.58	47.97	31.13	-27.63	-0.82	-1.30	-0.35	-2.57	0.44
PTH-K	9	-11.30	-7.64	-15.04	-7.86	-21.04	1.79	4.12	-0.99	-0.50
PTH-K	9	-11.28	-8.34	-15.27	-7.88	-20.01	1.66	2.52	0.37	0.25
PTH-K	9	-12.59	-7.47	-15.52	-7.71	-20.51	1.02	1.41	-1.73	2.11
PTH-K	9	-11.30	-9.58	-15.08	-7.31	-18.49	1.49	4.17	-0.54	0.58
PTH-K	9	-10.91	-8.77	-14.24	-7.38	-19.27	0.24	3.26	-1.46	1.37
PTH-L	10	-0.18	30.35	-1.40	0.48	15.76	5.36	2.45	-3.21	0.33
PTH-L	10	0.71	31.84	-0.90	0.36	16.03	5.75	2.78	-2.80	0.72
PTH-L	10	-0.09	31.08	-0.13	0.36	16.36	5.45	3.08	-3.39	1.49
PTH-L	10	1.26	31.32	-0.53	-0.04	16.46	5.24	2.60	-2.57	3.58
PTH-L	10	2.38	28.30	-3.60	2.06	15.40	4.58	3.95	-2.93	0.71
PTH-M	11	1.54	18.88	-6.29	7.82	-1.13	-1.47	-0.25	-1.53	-2.24
PTH-M	11	-0.15	16.61	-6.12	6.37	-1.11	-1.09	-0.47	-1.58	-1.92
PTH-M	11	1.00	18.09	-6.64	6.92	0.23	-1.95	-1.56	-3.22	-4.43
PTH-M	11	2.09	17.55	-5.60	7.30	-0.72	-1.71	0.79	-1.24	-1.82
PTH-M	11	1.70	15.80	-6.86	6.14	-0.79	-1.80	0.70	-1.87	-2.67
PTH-N	12	3.60	20.74	-10.35	5.55	1.04	-1.26	-3.95	4.66	-0.05
PTH-N	12	3.56	19.84	-9.90	4.97	0.75	-1.49	-4.08	4.42	-0.88
PTH-N	12	3.89	21.09	-9.67	4.79	0.90	-1.33	-3.51	3.24	-1.30
PTH-N	12	3.00	18.92	-9.74	2.16	0.72	-2.05	-5.74	3.32	-2.47
PTH-N	12	3.33	19.04	-9.77	4.11	2.42	-2.01	-5.11	3.44	-1.19
PTH-P	13	-9.02	-4.82	-21.59	-11.80	-6.67	8.12	-0.21	-2.36	-1.35
PTH-P	13	-11.11	-6.23	-20.31	-12.75	-6.57	8.49	0.67	-2.78	-0.01
PTH-P	13	-9.37	-5.67	-19.43	-12.43	-5.35	7.08	-0.78	-1.99	-0.98
PTH-P	13	-9.69	-7.53	-18.57	-13.69	-4.90	7.33	0.22	-1.40	0.49
PTH-P	13	-8.14	-5.84	-19.86	-13.04	-4.62	6.09	-2.13	-1.50	0.45
PTH-Q	14	9.11	23.58	-12.04	2.68	8.96	2.30	-1.22	3.27	0.05
PTH-Q	14	8.07	23.24	-10.36	4.29	11.41	1.87	-1.71	5.06	-1.53
PTH-Q	14	8.18	21.71	-11.54	0.47	8.25	2.94	-0.48	4.29	2.10
PTH-Q	14	10.22	23.54	-10.94	1.50	10.46	0.89	-1.23	1.44	-1.04
PTH-Q	14	9.45	21.31	-13.23	0.55	9.58	1.06	-2.27	2.17	-0.95
PTH-R	15	1.79	-9.09	-16.83	-17.65	2.05	0.01	-0.83	0.93	0.03
PTH-R	15	1.06	-8.53	-16.50	-18.27	1.32	1.33	-0.26	1.45	-1.20
PTH-R	15	2.20	-9.11	-15.24	-18.56	1.89	1.56	2.27	1.04	-2.11

PTH-R	15	2.39	-10.33	-14.90	-18.90	3.23	-0.40	-0.37	1.76	-0.78
PTH-R	15	2.62	-11.03	-15.46	-17.98	3.52	-1.10	-0.85	1.93	0.65
PTH-S	16	-56.84	-25.19	12.62	0.16	6.90	3.79	-2.89	1.76	1.44
PTH-S	16	-56.76	-26.39	12.99	-1.30	5.37	4.23	-2.79	2.71	1.91
PTH-S	16	-57.21	-26.52	13.87	0.45	8.56	3.87	-2.30	1.72	-0.56
PTH-S	16	-56.56	-26.92	14.22	-1.49	6.99	4.77	-1.54	3.35	-0.59
PTH-S	16	-56.68	-27.62	12.82	-0.17	8.31	3.81	-2.39	2.04	-0.17
PTH-T	17	-1.82	21.20	5.61	5.32	-11.84	-3.84	-0.91	3.19	2.65
PTH-T	17	-1.96	20.78	7.79	4.11	-14.65	-4.93	-1.54	4.65	0.60
PTH-T	17	-2.60	19.22	8.83	4.13	-13.94	-5.09	-0.79	1.93	0.46
PTH-T	17	-1.49	19.90	9.05	2.41	-14.45	-6.12	-0.89	1.65	0.41
PTH-T	17	-0.85	19.85	8.41	3.64	-15.21	-6.24	-1.89	2.68	1.56
PTH-V	18	40.71	-11.98	16.94	17.20	-9.37	8.71	-4.17	-1.91	-0.95
PTH-V	18	38.25	-9.69	15.65	15.86	-9.62	6.80	-3.06	-1.53	-2.64
PTH-V	18	38.44	-10.32	15.69	16.83	-7.65	8.62	-3.92	-3.52	1.19
PTH-V	18	34.64	-10.29	15.48	15.24	-7.48	5.71	-3.28	-2.81	2.01
PTH-V	18	38.03	-10.76	16.92	15.43	-10.79	5.49	-2.89	-1.64	1.19
PTH-W	19	-41.37	-18.34	6.82	8.27	-1.88	0.38	-0.40	1.11	2.68
PTH-W	19	-41.54	-17.32	5.17	7.02	-1.89	1.53	-0.95	-0.24	0.59
PTH-W	19	-42.38	-18.48	7.11	7.36	-0.62	0.99	-1.37	1.12	0.53
PTH-W	19	-40.90	-18.02	7.43	7.49	-1.23	1.06	-0.12	1.79	-0.06
PTH-W	19	-42.00	-19.09	7.68	7.36	-0.35	0.24	-1.60	1.23	1.61
PTH-Y	20	-5.96	15.90	-2.39	10.01	-4.09	-1.33	2.66	-0.63	-0.01
PTH-Y	20	-5.34	17.03	-1.52	9.46	-5.21	-1.95	1.90	-0.55	0.10
PTH-Y	20	-5.44	15.97	-1.51	10.01	-3.29	-1.44	1.91	0.40	0.52
PTH-Y	20	-6.95	15.60	-1.32	8.66	-2.06	-0.82	2.94	-0.78	-0.43
PTH-Y	20	-4.30	15.96	-2.27	8.68	-4.08	-1.55	2.83	-0.84	0.24

Table S3. LDA jackknifed classification matrix was obtained from the nine-element array against 20 PTH-amino acids.

	PTH-A	PTH-C	PTH-D	PTH-E	PTH-F	PTH-G	PTH-H	PTH-I	PTH-K	PTH-L	PTH-M	PTH-N	PTH-P	PTH-Q	PTH-R	PTH-S	PTH-T	PTH-V	PTH-W	PTH-Y	%correct
PTH-A	5	0	0	0	0	0	0	0	0	0	0	0	0	0	0	0	0	0	0	0	100
PTH-C	0	5	0	0	0	0	0	0	0	0	0	0	0	0	0	0	0	0	0	0	100
PTH-D	0	0	5	0	0	0	0	0	0	0	0	0	0	0	0	0	0	0	0	0	100
PTH-E	0	0	0	5	0	0	0	0	0	0	0	0	0	0	0	0	0	0	0	0	100
PTH-F	0	0	0	0	5	0	0	0	0	0	0	0	0	0	0	0	0	0	0	0	100
PTH-G	0	0	0	0	0	5	0	0	0	0	0	0	0	0	0	0	0	0	0	0	100
PTH-H	0	0	0	0	0	0	5	0	0	0	0	0	0	0	0	0	0	0	0	0	100
PTH-I	0	0	0	0	0	0	0	5	0	0	0	0	0	0	0	0	0	0	0	0	100
PTH-K	0	0	0	0	0	0	0	0	5	0	0	0	0	0	0	0	0	0	0	0	100
PTH-L	0	0	0	0	0	0	0	0	0	5	0	0	0	0	0	0	0	0	0	0	100
PTH-M	0	0	0	0	0	0	0	0	0	0	5	0	0	0	0	0	0	0	0	0	100
PTH-N	0	0	0	0	0	0	0	0	0	0	0	5	0	0	0	0	0	0	0	0	100
PTH-P	0	0	0	0	0	0	0	0	0	0	0	0	5	0	0	0	0	0	0	0	100
PTH-Q	0	0	0	0	0	0	0	0	0	0	0	0	0	5	0	0	0	0	0	0	100
PTH-R	0	0	0	0	0	0	0	0	0	0	0	0	0	0	5	0	0	0	0	0	100
PTH-S	0	0	0	0	0	0	0	0	0	0	0	0	0	0	0	5	0	0	0	0	100
PTH-T	0	0	0	0	0	0	0	0	0	0	0	0	0	0	0	0	5	0	0	0	100
PTH-V	0	0	0	0	0	0	0	0	0	0	0	0	0	0	0	0	0	5	0	0	100
PTH-W	0	0	0	0	0	0	0	0	0	0	0	0	0	0	0	0	0	0	5	0	100
PTH-Y	0	0	0	0	0	0	0	0	0	0	0	0	0	0	0	0	0	0	0	5	100
Total	5	5	5	5	5	5	5	5	5	5	5	5	5	5	5	5	5	5	5	5	100

Table S4. Identification of unknown PTH-amino acids samples using the optimized array.

Analyte	LDA scores									Results		
Unknown samples	(1)	(2)	(3)	(4)	(5)	(6)	(7)	(8)	(9)	Group	Identification	Verification
1	53.73	-35.47	13.23	2.64	-0.42	8.67	-5.76	2.36	-2.50	3	D	D
2	51.86	-26.83	2.11	-3.79	5.36	-2.19	0.16	0.48	-0.51	4	E	E
3	-35.71	-29.72	8.27	0.26	3.75	-2.69	6.28	-2.99	-1.53	2	C	C
4	-5.97	16.53	-1.87	10.08	-3.68	-0.98	3.12	-1.41	0.34	20	Y	F
5	-41.23	-18.22	7.47	8.41	-2.06	0.39	-1.78	0.43	0.42	19	W	W
6	34.75	-35.16	4.76	-3.88	2.24	3.91	0.06	3.31	2.16	7	H	H
7	-11.73	-9.40	-13.13	-7.24	-20.10	1.11	2.49	1.37	-0.45	9	K	K
8	1.30	-8.98	-15.78	-18.04	1.45	1.67	-0.03	2.32	-2.03	15	R	R
9	0.16	16.07	-5.37	6.50	-0.67	-0.66	0.60	0.20	-2.22	11	M	M
10	-12.48	-7.88	-17.60	-10.63	-4.90	7.28	-0.81	-2.36	-1.18	13	P	P
11	-57.88	-27.18	13.99	0.18	6.95	3.76	-2.46	1.73	1.50	16	S	S
12	-0.33	-9.87	-14.69	-17.08	1.82	1.50	0.83	1.24	-2.26	15	R	R
13	4.90	21.64	-2.86	9.81	0.18	-2.21	0.49	2.37	-2.42	1	A	A
14	-1.55	18.30	8.25	6.66	-11.93	-3.58	-2.55	4.56	1.77	17	T	T
15	39.43	-31.52	8.96	-1.21	0.31	0.59	-3.51	2.92	-1.59	3	D	H
16	7.02	21.12	-9.96	3.13	9.71	2.93	-1.23	6.70	2.36	14	Q	Q
17	-5.00	14.60	11.11	6.33	-15.10	-6.00	-2.37	2.56	2.65	17	T	T
18	-10.16	-8.10	-23.40	-7.97	-7.50	6.98	0.71	-2.79	1.33	13	P	K
19	0.70	30.97	0.28	2.17	16.96	5.57	2.55	-0.90	0.16	10	L	L
20	-7.47	17.13	1.57	18.88	4.67	-1.53	4.71	-0.98	1.42	5	F	F
21	-14.48	-12.32	-13.68	-1.52	-1.27	-3.18	-4.75	-2.39	0.73	6	G	G
22	5.22	20.92	-9.40	5.18	11.57	-0.30	-4.15	2.18	0.15	14	Q	Q
23	0.85	46.03	30.99	-22.21	-4.41	-3.30	-4.01	1.11	-2.06	8	I	I
24	-11.76	-8.67	-18.18	-11.94	-5.78	6.86	-1.92	-0.97	0.94	13	P	P
25	-3.42	11.85	-5.37	5.42	-0.86	-1.79	-1.33	-1.00	1.03	11	M	M
26	-36.47	-31.01	10.98	-0.02	3.13	-3.46	5.24	-0.33	-2.74	2	C	C
27	-40.91	-17.77	7.04	8.02	-2.09	1.33	-1.21	1.10	-0.42	19	W	W
28	3.25	19.43	-7.96	6.27	2.46	-1.98	-4.32	5.43	-1.78	12	N	N
29	-5.34	15.86	-0.73	10.92	-3.26	-1.84	1.57	1.17	-0.49	20	Y	Y
30	-2.56	27.46	-0.09	3.67	17.36	3.00	0.71	-5.55	4.27	10	L	L
31	-13.99	-11.08	-14.32	-0.18	1.83	-3.62	-5.85	-4.27	-1.61	6	G	G
32	-42.76	-19.05	8.61	8.45	-1.06	0.94	-1.54	1.46	0.03	19	W	W
33	45.54	-11.29	17.54	17.89	-11.85	13.19	-4.64	0.98	-4.11	18	V	V
34	2.92	19.98	-3.32	9.26	-1.05	-1.87	1.40	0.65	0.32	1	A	A
35	-4.81	18.37	0.82	14.12	-1.60	-2.06	2.67	-0.66	-0.13	5	F	Y
36	32.55	-35.06	6.25	-4.57	1.88	2.73	0.24	4.85	1.32	7	H	H
37	-12.44	-8.50	-17.36	-10.82	-4.23	6.44	-1.93	-2.00	-0.20	13	P	P
38	-1.47	41.69	29.28	-17.97	-0.30	-3.08	-3.67	0.95	2.32	8	I	I
39	1.21	17.45	-7.25	6.75	2.65	-3.66	-5.60	2.32	0.50	12	N	N
40	-2.39	20.76	6.54	14.70	-11.06	-8.85	-0.10	3.43	0.98	17	T	T
41	-15.43	-12.21	-12.78	-5.65	-16.05	0.92	3.27	-1.96	1.65	9	K	K
42	-0.19	-10.22	-14.90	-17.44	1.94	1.36	1.03	1.02	-2.19	15	R	R
43	-15.31	-10.73	-14.18	-0.32	2.27	-2.16	-5.49	-3.18	-3.83	6	G	G
44	-2.79	27.22	0.75	4.70	17.25	3.25	1.70	-4.43	3.31	10	L	L
45	4.51	21.34	-3.33	8.90	-0.14	-2.95	-0.92	1.28	-0.76	1	A	A
46	-11.03	12.84	0.44	16.50	0.41	-0.91	5.60	-0.45	5.07	5	F	F
47	43.63	-9.49	14.78	17.22	-12.62	10.48	-6.35	-0.03	-0.43	18	V	V
48	-3.92	14.45	-3.55	9.44	1.17	-2.52	-1.51	-2.11	-0.67	20	Y	M
49	36.47	-26.67	3.88	-2.53	5.13	-2.58	-3.55	1.46	-0.87	7	H	D
50	3.55	18.90	-8.15	4.15	11.76	-0.13	-2.31	-0.18	0.63	14	Q	Q
51	41.17	-12.35	18.49	18.90	-13.50	13.57	-3.90	-1.93	-1.62	18	V	V
52	9.69	21.59	-10.28	2.66	9.68	0.89	-0.95	5.03	-1.54	14	Q	N
53	-56.86	-26.73	13.92	0.80	6.40	3.77	-2.38	2.36	-0.84	16	S	S
54	-35.90	-31.38	12.30	0.35	1.77	-4.41	6.09	-0.80	-2.53	2	C	C
55	-3.88	43.42	35.76	-16.44	-6.64	-8.83	-4.01	-5.07	3.90	8	I	I
56	50.01	-29.44	2.55	-3.35	4.44	-0.21	-1.97	1.18	0.81	4	E	E
57	43.17	-24.40	-0.87	-8.00	5.92	-6.89	2.29	0.16	-0.22	4	E	E
58	-58.94	-28.84	14.55	-0.82	6.95	4.77	-1.39	3.17	0.29	16	S	S
59	45.18	-35.68	13.00	3.20	-0.63	6.83	-8.51	4.74	-3.51	3	D	D
60	-10.05	12.53	2.34	11.52	-4.17	-3.47	1.15	-1.60	0.07	20	Y	Y

Complex titrations and determination of K_{SV} constants

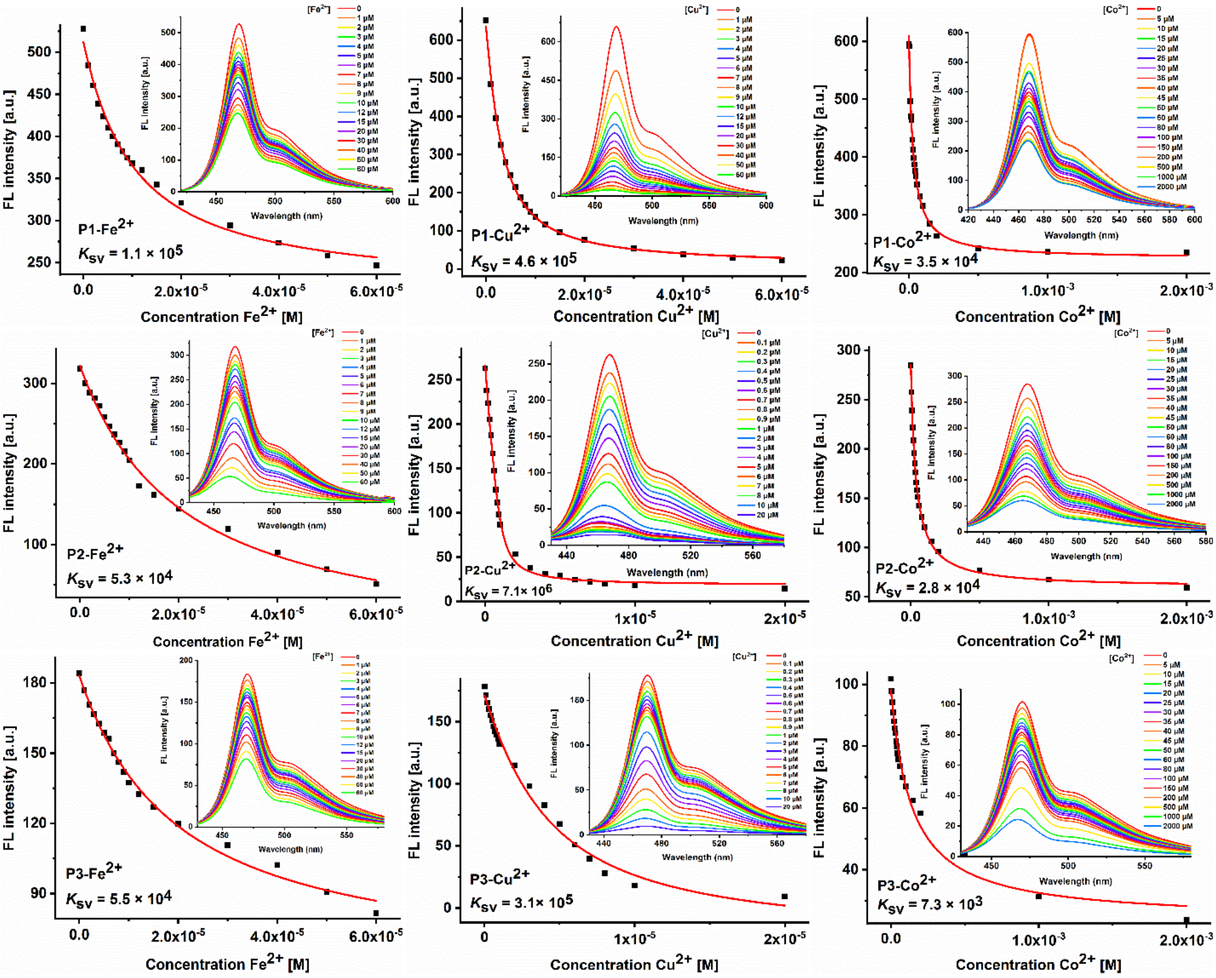

Figure S12. Stern–Volmer plots for fluorescence quenching of PPEs (2 μM) titrated with metal cations (Fe^{2+}, Cu^{2+} and Co^{2+}) in DMSO/H_2O (1:1). The inset show the emission of quenching data and K_{SV} values.

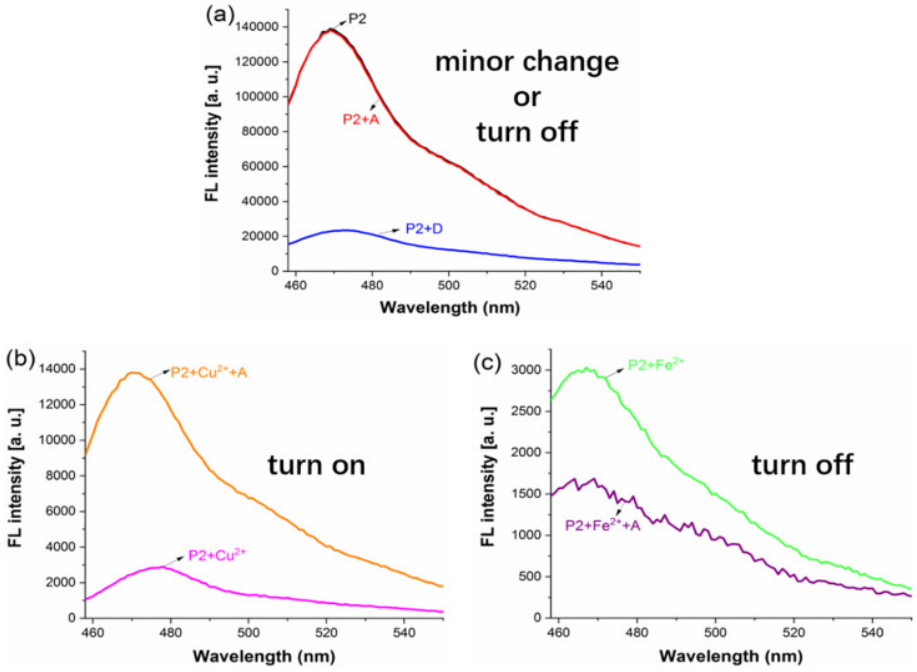

Figure S13. (a) Emission spectra of **P2** (2 μM) upon interaction with PTH-amino acids; (b, c) Emission spectra of **P2** upon interaction with metal ions/ PTH-amino acids. The spectra were measured in DMSO/H_2O (1:1). PPE-**P2**, PTH-amino acids (A, D) and metal ions (Fe^{2+}, $Cu^{2+)}$ were selected as examples to illustrate the overall changes in fluorescence intensity. 'PTH' was omitted for clarity.

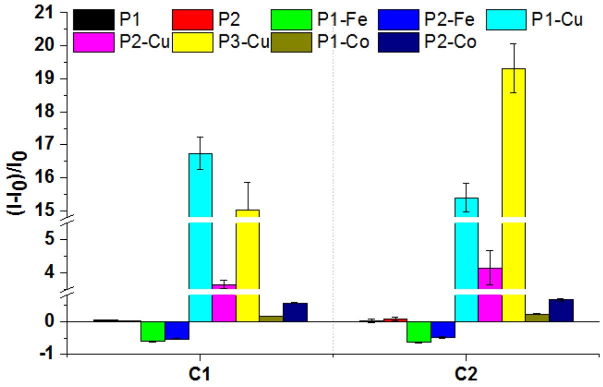

Figure S14. Fluorescence response pattern ($(I - I_0)/I_0$) of nine optimized elements treated with two degradation residues in DMSO/H_2O (1:1) after incubation for 2 h at room temperature. Each value is the average three independent measurements and each error bar shows the standard error of these measurements.

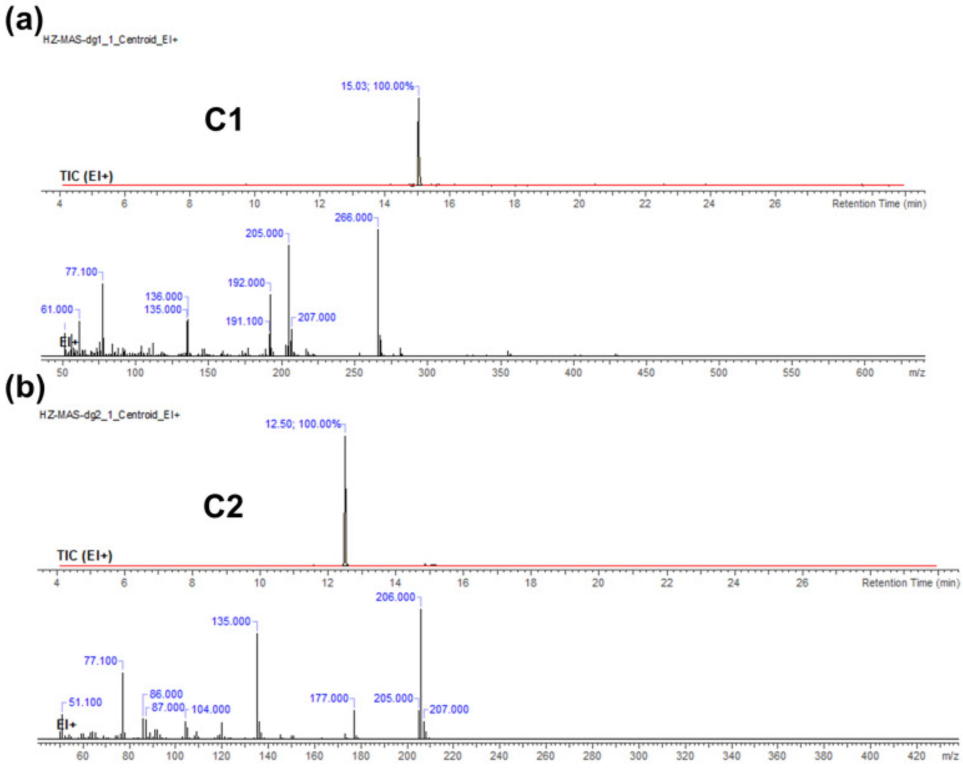

Figure S14. GC-MS spectrum of degradation residues generated from the two cycles.

Table S5. Degradation residues identification by GC-MS analysis.

Cycle	Degradation residue	Yield	Retention time (min)	m/z	Identified by GC-MS	Amino acid
1º	C1	80%	15.03	266	PTH-M	Met
2º	C2	62%	12.50	206	PTH-A	Ala

3. Detailed procedure for Edman degradation

Stepwise degradation for oligopeptide was performed by a modification of a previously described method.[2]

3.1. Preparation of the PTC-peptide

To a glass centrifuge tube, 28.9 mg of Met-Ala-Ser was added and fully dissolved in 2 mL pyridine-water (1:1, v/v, pH ca. 8.8). Under nitrogen atmosphere, 200 μL phenyl isothiocyanate (PITC) was added to the solution of peptide. The reaction mixture was vigorously stirred (1000 rpm) for 30 minutes in a 50 °C water bath. After the completion of the reaction, the mixture was cooled down to 0 °C. The PTC-derivative was thoroughly washed and extracted using benzene (5-8 times) and the phases were separated by centrifuge for 3 min at 3500 rpm. The excess PITC and side products in upper phase was removed and the water phase was concentrated under a stream of nitrogen, and then dried in vacuum.

3.2 Cleavage of the PTC-peptide

Under nitrogen atmosphere, the dried PTC-peptide was treated with anhydrous TFA (300 μL). The cleavage reaction was carried out in a water bath (50 °C, 10 min.). The TFA was removed using a flow of nitrogen and then dried under vacuum. The thiazoline derivative (ATZ-amino acid) was extracted from the remaining peptide with ethyl acetate/H_2O (2:1, v/v, 3 mL). The remaining peptide in water phase (1 mL) was coupled immediately for next round degradation (see **3.1**) or kept in the freezer. The ATZ-amino acid in the ethyl acetate phase was flushed with N_2 and dried under vacuum.

3.3 Transformation of the ATZ-amino acid

Under nitrogen atmosphere, the dried ATZ-amino acid was dispersed in 300 μL 1N HCl and the conversion reaction was carried out in a water bath (80 °C, 10 min.). After the completion of the reaction, the mixture was cooled down to 0 °C. The mixture was extracted 3 times with ethyl acetate and the organic phase containing PTH-amino acid was collected after centrifugation. The ethyl acetate layer was washed with saturated sodium bicarbonate solution followed by washing with

brine, then flushed with N_2 and dried under vacuum vacuum. The degradation residue was recrystallized from EtOH to afford final product for the sensing experiments.

In the first cycle, the residue **C1** was obtained with a yield of 80%. The second degradation was performed according to **3.1-3.3** and the residue **C2** was obtained with a yield of 62%. The residues were dissolved in DMSO and diluted using water to make a stock solution (2 mg/mL) for sensing experiments and the final concentration was controlled at 1 mg/mL.

4. References

[1] a) I.-B. Kim, R. Phillips and U. H. F. Bunz, *Macromolecules* **2007**, *40*, 5290-5293; b) B. Wang, J. Han, M. Bender, K. Seehafer and U. H. F. Bunz, *Macromolecules* **2017**, *50*, 4126-4131. c) J. Han, C. Ma, B. Wang, M. Bender, M. Bojanowski, M. Hergert, K. Seehafer, A. Herrmann, U. H. F. Bunz, *Chem* **2017**, *2*, 817-824; d) J. Han, M. Bender, S. Hahn, K. Seehafer, U. H. F. Bunz, *Chem. Eur. J.* **2016**, *22*, 3230–3233; e) J. Han, B. Wang, M. Bender, K. Seehafer, U. H. F. Bunz, *ACS Appl. Mater. Interfaces* **2016**, *8*, 20415–20421.

[2] P. Edman, *Acta Chem. Scand.* **1950**, *4*, 283-293.
